# A Latent Class Analysis of Parental Alcohol and Drug Use: Findings from the Avon Longitudinal Study of Parents and Children

**DOI:** 10.1016/j.addbeh.2019.106281

**Published:** 2020-05

**Authors:** Emily Lowthian, Graham Moore, Giles Greene, Sara Madeleine Kristensen, Simon C. Moore

**Affiliations:** a Centre for the Development and Evaluation of Complex Interventions for Public Health Improvement, School of Social Sciences, Cardiff University, Cardiff, United Kingdom; b Crime and Security Research Institute, Friary House, Greyfriars Road, Cardiff, United Kingdom; c Violence Research Group, School of Dentistry, Cardiff University, Cardiff, United Kingdom; d Department of Health Promotion and Development, University of Bergen, 5020, Bergen, Norway; e Division of Population Medicine, Cardiff University, Cardiff, United Kingdom

**Keywords:** Parent, Substance, Latent class analysis, ALSPAC, Drugs, Alcohol

## Abstract

Previous measures of parental substance use have often paid limited attention to the co-occurrence of alcohol and drugs, or to the between-parent dynamics in the use of substances. These shortcomings may have important implications for our understandings of the relationship between parental substance use and child wellbeing. Using data from the Avon Longitudinal Study of Parents and Children, a UK community-based cohort study from 1990 onwards (*n* = 9,451), we identified groups of parental substance use using latent class analysis. The 4-class solution offered the best fit, balancing statistical criteria and theoretical judgement. The results show distinct classes across the range of parental substance use, including very low users, low users, moderate users and heavy users. These classes suggest that substance use patterns among mothers are somewhat mirrored by those of their partners, while heavy use of alcohol by mothers and their partners is related to increased mothers drug use. We suggest that studies that investigate the effects of parental substance use on child wellbeing should pay greater attention to the dynamics of substance use by parental figures.

## Highlights

• We identified four parental substance use classes using quantity-frequency measures.
• Heavy use of alcohol formed a class which also included a large proportion of drug users.
• Mothers’ and their partner’s tended to have similar patterns of substance use behaviours.

## 1 Introduction

A growing body of research indicates that parental substance use is detrimental to children’s key developmental outcomes and wellbeing (Kuppens et al., 2019, McGovern et al., 2018, Rossow et al., 2016, Velleman and Templeton, 2007). It is associated with an increase in a child’s risk of injury (Crandall et al., 2006, Paranjothy et al., 2018, Raitasalo et al., 2015, Woodside et al., 1993), internalising symptoms (Chassin et al., 1999, Kelley et al., 2017, Lee and Cranford, 2008), externalising symptoms (El-Sheikh and Flanagan, 2001, Hussong et al., 2010), and lower educational outcomes, including attainment (Berg et al., 2016, Mangiavacchi and Piccoli, 2018, Torvik et al., 2011). Estimates suggest that up to 30% of children in the UK have lived with a problem drinker, including binge, hazardous or dependent drinkers (Manning, Best, Faulkner, & Titherington, 2009). Up to 8% of children have lived with an adult who used illicit drugs, 3% of whom were dependent users (Manning et al., 2009). Those who take drugs can also use alcohol at harmful levels, and estimates suggest that 4% of children lived with a problem drinker who also used drugs in the past year (Manning et al., 2009). Furthermore, recent estimates indicate that 20% of those in treatment for substance misuse (alcohol and/or drugs) lived with children, and a further 31% were parents who did not live with their children (Public Health England, 2018).

Our understanding of how substance use affects child health and wellbeing has been hampered by inconsistency in the way in which substance use is conceptualised and quantified in epidemiological surveys. Some research focuses only on clinically relevant levels of consumption according to DSM-IV criteria, while others use quantity-frequency measures, or screening tools such as the Alcohol Use Disorders Identification Test (AUDIT), and therefore identify individuals who are potentially ‘problem’ drinkers (Saunders, Aasland, Babor, Fuente, & Grant, 1993). The focus on clinical levels of substances has been helpful for understanding the effects on children who are at greatest risk. However, such measures fail to capture variation in drinking at sub-clinical levels that may also have important impacts on child wellbeing. Nevertheless, the measures of subclinical substance use have limitations, including a common focus on either alcohol *or* illicit drug use, which neglects the complexities apparent in real-world consumption; notably, poly-drug use (EMCDDA, 2002)*.*

Maternal and paternal effects are typically considered separately (e.g. Torvik et al., 2011). This neglects the combined effect of parents’ drug and alcohol use on child wellbeing and fails to account for the family as a system. A holistic approach is crucial when an individual functions within a family, and behaviour is not fully understood without taking into account the dynamics of the family system (Lander, Howsare, & Byrne, 2013). This is relevant when individual alcohol use is associated with the behaviour of their relatives (Rosenquist, Murabito, Fowler, & Christakis, 2010). If we are to understand parental substance use, it is necessary to understand the complexity of parental substance use, acknowledging that it goes beyond alcohol *or* illicit drug use, and maternal *or* paternal effects. The purpose of this research is to explore parental substance use, adjusting for mothers and their partner’s use of both alcohol *and* illicit drugs. From this, we aim to add to the growing literature on substance use generally (Agrawal et al., 2007, Evans-Polce et al., 2016), whilst offering a unique finding in terms of parental substance use behaviours and the dynamics between them. This is undertaken using data from the Avon Longitudinal Study of Parents and Children (ALSPAC), a community-based cohort study in the UK. We then endeavour to consider these findings in terms of child wellbeing and the implications surrounding this.

## 2 Materials and methods

### 2.1 Data and participants

ALSPAC recruited participants who were women, pregnant and residents of the former administrative county of Avon between 1990 and 1992 (Boyd et al., 2013). Recruitment occurred in two phases due to a small number of children being later identified as eligible for the study but missed the first recruitment stage. The first phase recruited participants by advertising and postpartum by clinical staff (Boyd et al., 2013). This recorded 14,541 pregnancies, 674 of which were excluded due to miscarriage and stillbirth, resulting in 13,867 children being eligible at birth. ALSPAC estimate that 82.6% of the eligible pregnancies were enrolled in the first phase of recruitment. The second phase of recruitment was the introduction of ‘Focus@7′ which was conducted when the child was seven years of age. This second recruitment drive increased the number of eligible pregnancies enrolled in ALSPAC to 15,274 (Boyd et al., 2013, Fraser et al., 2013). As the analyses reported here are measures collected when the children were three years of age, only children born in the first phase of recruitment were eligible. The final sample included 13,761 women (13,867 pregnancies). These women, their partners, and children have received regular questionnaires since recruitment and continue to be contacted as of the publication date. The study website contains details of all the data that is available through a fully searchable data dictionary and variable search tool: www.bristol.ac.uk/alspac/researchers/our-data/.

### 2.2 Attrition and missing data

Since recruitment, women have left the study due to the death of the child, becoming untraceable, or withdrawing from the study (Boyd et al., 2013). In addition, some women, and their partners and children, did not respond to all questionnaires. This attrition and non-response has led to a steady decrease in the eligible sample over time and an over-representation of more affluent, white ethnic groups compared to the national population (Boyd et al., 2013, p. 124). Due to this, we used earlier data from the mother, when the child was three years of age as around three-quarters of the initial sample were eligible for the study at this time (*n* = ~10,000) (Fraser et al., 2013). Our analytical sample was 69% (*n* = 9,451) of mothers who were eligible since the first phase of recruitment (*n* = 13,761). Full Information Maximum Likelihood was used to account for missing data and all available cases were used in analysis.

### 2.3 Ethical procedure

Ethical approval was obtained from the ALSPAC Ethics and Law Committee, the Local Research Ethics Committees and Cardiff University’s School of Social Sciences. Informed consent for the use of data collected using questionnaires and in clinics was obtained from participants.

### 2.4 Measures

#### 2.4.1 Mothers’ alcohol use

Mothers’ self-reported alcohol use was collected when their child was 3 years and 11 months of age. The postal questionnaire used a one-week diary method (Monday through to Sunday) with respondents reporting the number of glasses of alcohol consumed in the past seven days only. A glass was defined as 25 ml of spirits, ½ pint (284 ml) of beer or cider, or a 125 ml wine glass of wine. The diary used categories of “beer, lager or cider (number of ½ pints)”, “wine (number of glasses)”, “spirits (number of single pub measures)”, “other alcohol drinks (number of glasses or measures)”, and “low alcohol drinks (number of glasses or ½ pints)”. The number of glasses was totalled for each day of the week.

#### 2.4.2 Partners’ alcohol use

Partners’ alcohol use was collected from the mother when the child was three years and 11 months of age. While separate partner data existed, response rates (*n* = 4,788, 35%) were not sufficient for it to be used as a primary measure. Mothers’ estimate of their partner’s alcohol use was validated against the partners’ own report for the same question (*r_s_* = 0.73, *p* < 0.05, *n* = 4,386). The mother answered the question, “How many days in the past month do you think he had the equivalent of two pints of beer, four glasses of wine or four pub measures of spirit?”. The responses were “everyday”, “>10 days”, “5–10 days”, “3–4 days”, “1–2 days”, “none”, and “don’t know.” Answers of “don’t know” and “no partner” were recoded as missing for analysis. Although the he/him pronouns were used, gender was not stipulated in the questionnaire invitation (Golding, Pembrey, Jones, & The ALSPAC Study Team, 2001).

#### 2.4.3 Drug use

Mothers’ drug use was collected when the child was three years and 11 months of age. It was aggregated to form a binary response using sub-questions from the question “In the past year how often have you taken or used the following?”. The sub-questions included the use of cannabis, tranquilisers, amphetamines, or other stimulants such as heroin, methadone, crack or cocaine. Binary response was superior to more detailed responses due to low response rates for individual, more detailed drug use, which would have likely caused boundary errors during estimation. The responses of “every day”, “often” and “sometimes” formed the binary category “yes”, and the response “not at all” formed the binary category “no.” Partner self-report drug use was not used due to the low number of observations (*n* = 4,823, 35%), and the mother was not asked a question regarding their partner’s drug use.

### 2.5 Statistical analysis

Data were managed in Stata 15.2 (StataCorp., 2017) and converted to Mplus version 8.2 (Muthén & Muthén, 2017) for analysis. First, the confirmatory factor analysis of mothers’ alcohol use was conducted to ensure that the data fitted the model and could be used in a latent class analysis. Second, the latent class analysis was fitted using the variables of mothers’ alcohol use (latent variable), partners’ alcohol use, and mothers’ drug use.

#### 2.5.1 Confirmatory factor analysis

A latent variable of the mothers’ alcohol use was created using confirmatory factor analysis and using seven variables that described the total number of glasses of alcoholic beverages on each day of the week. This was undertaken due to a lack of independence across days. As with similar research, a negative binomial distribution was used to estimate the latent variable (Horton et al., 2007, Iwamoto et al., 2011, Lewis et al., 2009, Neal and Simons, 2007). The maximum likelihood robust estimator was used, and the χ^2^ and factor loadings (above ± 0.4) were used as model fit criteria (Brown, 2015). Once the fit criteria were achieved, the latent variable of mothers’ alcohol use was used in the latent class analysis in a one-step method.

#### 2.5.2 Latent class analysis

Latent class analysis, a derivative of factor analysis, was used to explore unobserved constructs in observed data (Melendez-Torres et al., 2018) and therefore the presence of underlying classes in the parental substance use variables were identified. This form of analysis facilitates an understanding of substance use in context, going beyond one-dimensional definitions (i.e. alcohol *or* drugs) and mutually exclusive conceptualisations (i.e. maternal *or* paternal). The variables used included mothers’ alcohol use, constructed as a latent variable, and the manifest variables of partners’ alcohol use and mothers’ drug use.

A maximum likelihood robust estimator was used for the latent class analysis; the number of classes were determined by testing model fit for two, three, four, five and six latent classes. The classes were assessed according to “the model that best balanced interpretability and fit” (Melendez-Torres et al., 2018, p. 160) and on five statistical criteria: Akaike Information Criterion (AIC), Bayesian Information Criterion (BIC), Entropy, a measure whereby “0% indicates very poor certainty in classification and 100% indicates perfect certainty” (Melendez-Torres et al., 2018, p. 160), the Vuong-Lo-Mendell-Rubin likelihood ratio test (VLMR-LRT) and the Lo-Mendell-Rubin adjusted likelihood ratio test (LMR-LRT), as recommended by Geiser (2013). The bootstrap likelihood ratio difference test that Mplus offers was not used due to the high computational cost.

## 3 Results

The analytic sample consisted of 9,451 mothers. The graphs for the latent class analysis are presented separately for each variable due to differences in their measurement.

### 3.1 Sample demographics

Table 1 presents the demographic characteristics of the sample (totals<9,451 is due to missing data). Mothers’ age was taken when the child was delivered, mothers’ ethnicity and education was taken at 32 weeks gestation, and weekly family income was collected when the child was three years and 11 months of age. Table 2 shows the variables used in the latent class analysis. All variables are reported by the mother. Percentages were derived from the full eligible sample (*n* = 13,761).

**Table 1:** Demographic characteristics of analytic sample.

Demographics of sample used in analysis *(% of n = 13,761)*	N (%)
**Number of mothers with partner alcohol data***(58%)*	8,019
**Whether partner lives in the home***(n = 9,451, 69%)*	
Yes	8,384 (89%)
No (including mothers who have no partner)	1,067 (11%)
**Number of mothers without partners**	653 (5%)
**Age of mother at child’s delivery***(not available due to disclosure)*	
18 years and under	1%
19 – 30 years	65%
31 – 40 years	33%
41 years and above	1%
**Mother’s ethnicity***(n = 9,112, 66%)*	
White	8,956 (98%)
Black/ethnic minority	156 (2%)
**Mother’s qualifications***(n = 8,739, 64%)*	
None	312 (4%)
Certificate of Secondary Education	781 (9%)
Vocational/Apprenticeship/C&G intermediate	803 (9%)
O-level	3,255 (37%)
A level/State enrolled/registered Nurse/C&G Final or Full Technical	2,254 (26%)
Degree level	1,334 (15%)
**Weekly family income***(n = 8,491, 62%)*	
Less than £100	657 (8%)
Between £100 - £199	1,330 (16%)
Between £200 - £299	2,233 (26%)
Between £300 - £399	1,875 (22%)
Greater than £400	2,396 (28%)

**Table 2:** Variables used in analysis.

Variables for analysis	N (%)	Mean	SD
**Mothers’alcohol use***(n = 9,449, 69%)*			
Monday	9,449	0.43	1.16
Tuesday	9,449	0.45	1.07
Wednesday	9,449	0.50	1.18
Thursday	9,449	0.51	1.19
Friday	9,449	0.88	1.62
Saturday	9,448	1.29	2.06
Sunday	9,449	0.74	1.48
**Partners’alcohol use***(n = 8,019, 58%)*			
None	1,322 (16%)		
1 – 2 days	1,455 (18%)		
3 – 4 days	1,616 (20%)		
5 – 10 days	1,960 (24%)		
> 10 days	1,218 (15%)		
Everyday	448 (6%)		
**Mothers’drug use***(n = 9,415, 68%)*			
No	8,901 (95%)		
Yes	514 (5%)		

### 3.2 Mothers’ alcohol use – latent variable

Confirmatory factor analysis of mothers’ alcohol use produced acceptable model fit (*n* = 9,449, χ^2^ (77,823) = 45,010.89, *p* ≈ 1.00); note, some extreme values were deleted, and large values were truncated at the value of four in this estimation. Most of the factor loadings were excellent, and average factor loadings were 0.76, with Friday, Saturday, and Sunday drinking loading slightly lower (0.71, 0.54, and 0.63 respectively); this was expected when Monday drinking was the scaling variable (1.00). This model was accepted for use in the latent class analysis.

### 3.3 Final model decision

The 4-class solution showed the best model fit overall as it had low AIC and BIC values, acceptable entropy, acceptable class probabilities, and statistically significant (*p* < 0.05) LRT values, suggesting that it was better than the 2- and 3-class solution; see Table 3 for full statistical information on each class. Although the AIC and BIC was not the lowest value in the 4-class solution, the difference between the 4-class and 5-class solution BIC was very small (<5.00) and the 6-class solution was a larger number; the 4-class solution showed larger decreases compared to the 2 and 3-class solution. LRT tests suggested that a 2, 3, 4 and 5-class solution was acceptable (*p* < 0.05) but not a 6-class solution (*p* = 0.36 and 0.37). The highest entropy was found in the 2-class model (0.78), but the 4-class model still showed an acceptable entropy value (0.74), whereas the 5-class and 6-class model were borderline or less than adequate (0.68/0.70) (Wang, Deng, Bi, Ye, & Yang, 2017). Alongside the poor entropy, the 5-class and 6-class solution had classes with less than adequate classification accuracy (<80%) (Rost, 2006) whereas the 2-, 3- and 4-class solutions were all adequate. In addition, the 6-class solution had many thresholds set at extreme values, and could not replicate the best log-likelihood, so the model may not be valid due to local maxima. As a result, the 4-class solution offered the most detailed information on parental substance use whilst balancing statistical criteria.

**Table 3:** Latent class analysis statistical criterions, the 4-class solution accepted in bold.

	2-class	3-class	4-class	5-class	6-class
AIC	162,975.51	160,272.24	**159,596.63**	159,536.54	159,526.26
BIC	163,218.75	160,572.70	**159,954.32**	159,951.46	159,998.41
Proportions	52% (*n* = 4934)	50% (*n* = 4746)	**38% (*n* = 3584)**	33% (*n* = 3086)	32% (*n* = 3041)
	48% (*n* = 4517)	29% (*n* = 2735)	**30% (*n* = 2857)**	27% (*n* = 2559)	27% (*n* = 2558)
		21% (*n* = 1970)	**27% (*n* = 2591)**	27% (*n* = 2544)	26% (*n* = 2418)
			**4% (*n* = 420)**	11% (*n* = 1078)	13% (*n* = 1196)
				2% (*n* = 184)	2% (*n* = 235)
					0% (*n* = 2)
Entropy	0.78	0.77	**0.74**	0.68	0.70
Classification accuracy*	94%	91%	**86%**	80%	79%
	93%	92%	**84%**	88%	88%
		88%	**89%**	71%	68%
			**85%**	72%	71%
				82%	82%
					100%
VLMR LRT	*p* < 0.05	*p* < 0.05	***p* < 0.05**	*p* < 0.05	*p* = 0.36
LMR LRT	*p* < 0.05	*p* < 0.05	***p* < 0.05**	*p* < 0.05	*p* = 0.37

*Average latent class probabilities for most likely latent class membership

### 3.4 Sample proportions and means of 4-class solution

**Fig. 1:**
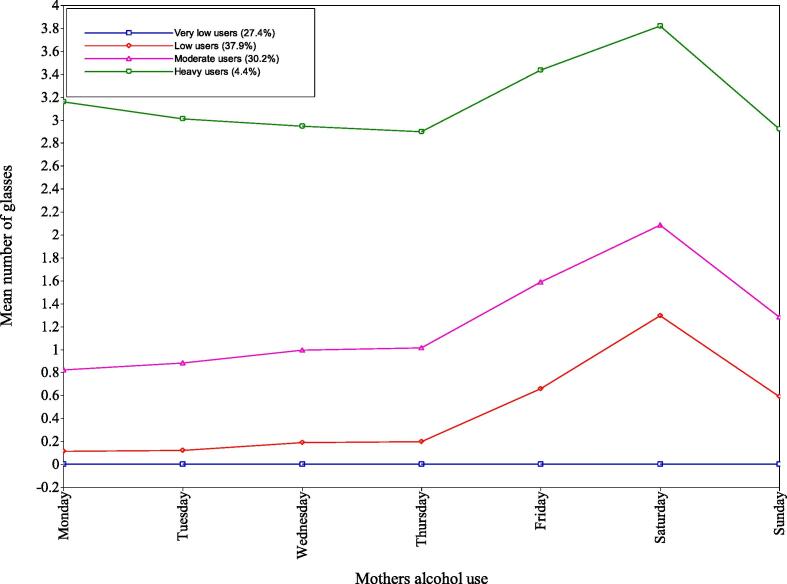
Mothers’ alcohol use - mean number of glasses for each class by day of the week.

**Fig. 2:**
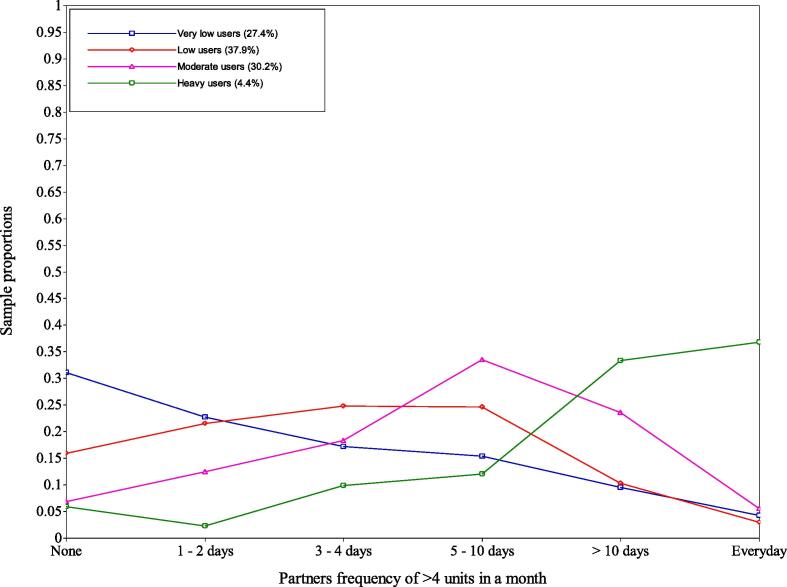
Sample proportions of partners’ alcohol use.

**Fig. 3:**
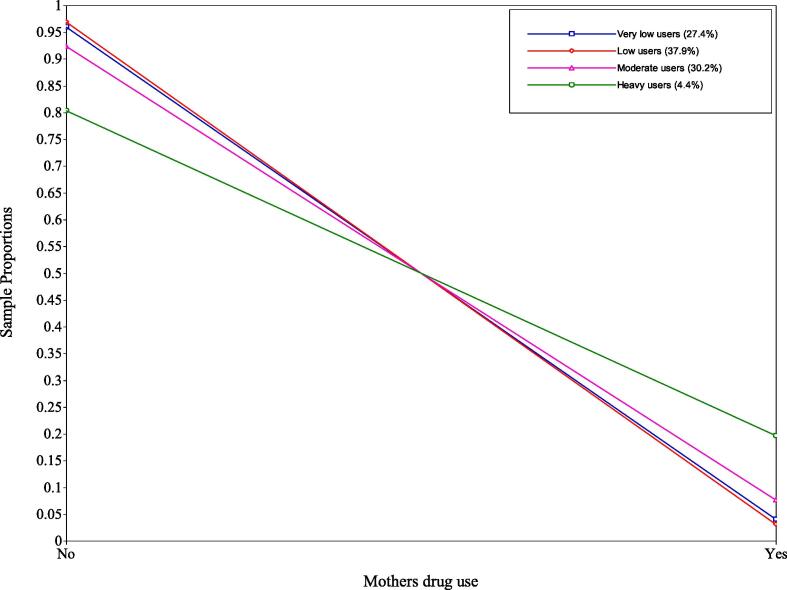
Sample proportions of mothers’ drug use.

The 4-class solution showed distinct classes in terms of mothers’ alcohol use (see Fig. 1), partners’ alcohol use (see Fig. 2), and mothers’ drug use (see Fig. 3). They were as follows: Class 1 - very low users, Class 2 – low users, Class 3 – moderate users, and Class 4 – heavy users.

*Class 1 – very low users.* This class comprised 27.4% of the respondents, the second smallest class. For mothers’ alcohol use, the latent variable was fixed at zero for all days due to very small parameters on the logit scale, this was cross-checked with Mplus support (Muthén & Muthén, 2017). It also contained the highest proportion of partners drinking more than four units on “none” (31%) or “1–2 days” (23%). However, 14% of the partners were in the “>10 days” (10%) and “everyday” (4%) category which shows differences compared to the mothers’ alcohol use. For mothers’ drug use, only 4% answered “yes” to this question.

*Class 2 - low users.* This class included the largest number the respondents (37.9%). For mothers’ alcohol use, these mothers averaged a small number of ‘glasses’ on Monday to Thursday (between 0.11 and 0.20 glasses). However, during Friday, Saturday, and Sunday these mothers increased their use (between 0.59 and 1.29 glasses) with Saturday use being the highest (1.29 glasses). This class had higher proportions of partners drinking more than four units on “3–4 days” (25%), “5–10 days” (25%) and “1–2 days” (22%). Like the very low users class, 13% of partners drank on “>10 days” (10%) and “everyday” (3%) showing a small discrepancy to the mothers’ alcohol use. For mothers’ drug use, only 3% answered “yes” to this question.

*Class 3 - moderate users.* This class included the second largest number of respondents (30.2%). For mothers’ alcohol use, mothers averaged near one glass between Monday and Thursday (0.82–1.01). However, during Friday, Saturday and Sunday these mothers increased their use (between 1.28 and 2.08 glasses) with Saturdays use being the highest (2.08 glasses). This class had the highest proportion of partners drinking more than four units on “5 – 10 days” (34%), and also had higher proportions of partners drinking on “> 10 days” (24%) and “3 – 4 days” (18%); few drank “everyday” (6%). For mothers’ drug use, 8% answered “yes” to this question, which suggests some poly-drug use in this class.

*Class 4 – heavy users.* This class included the smallest number of respondents (4.4%). Mothers’ alcohol use averaged around three glasses throughout the week, with Saturday having the highest value (3.82) and Thursday having the lowest (2.90). This consistent use throughout the week is distinct to other classes. This class had the highest proportion of partners drinking more than four units “everyday” (37%) and “> 10 days” (33%); conversely, it had the lowest proportion of “none” (6%), “1 – 2 days” (2%), “3 – 4 days” (10%) and “5 – 10 days” (12%). For mothers’ drug use, 20% answered “yes” to this question, the highest of all classes.

### 3.5 Demographics of latent classes

The demographics for each class are presented in Table 4. Mothers’ with no partners living in the home had higher percentages of being in the very low users class (37%) compared to mothers with partners living in their home (30%), and had lower percentages of being in the low users class (29% compared to 37%); mothers were similar for moderate use and heavy use. Mothers who had higher qualifications were more likely to be in the moderate class, and low users class, compared to mothers who had less qualifications, who were more likely to be in the very low users class; the heavy class fluctuated, with the opposing ends of the qualifications variable (None and Degree) having the highest proportions (6% and 5% respectively). Household income shows a similar pattern, which is expected since they are both measures of socioeconomic status. Younger mothers had higher percentages of being in the very low users class, and older mothers were more likely to be in the moderate and heavy classes.

**Table 4:** Demographics of each latent class.

	Very low users	Low users	Moderate users	Heavy users
**Number of mothers with partner alcohol data***(n = 8,019)*	2,310 (29%)	3,026 (38%)	2,383 (30%)	300 (4%)
**Whether partner lives in the home***(n = 9,451)*				
Yes	2,519 (30%)	3,133 (37%)	2,436 (29%)	296 (4%)
No (including mothers who have no partner)	399 (37%)	306 (29%)	310 (29%)	52 (5%)
**Age of mother at child’s delivery***(not available due to statistical disclosure)*				
18 years and under	48%	27%	22%	4%
19 – 30 years	34%	37%	26%	3%
31 – 40 years	24%	35%	36%	5%
41 years and above	35%	25%	33%	6%
**Mothers’qualifications***(n = 8,739)*				
None	154 (49%)	81 (26%)	58 (19%)	19 (6%)
Certificate of Secondary Education	323 (41%)	254 (33%)	183 (23%)	21 (3%)
Vocational/Apprenticeship/C&G intermediate	319 (40%)	305 (38%)	160 (20%)	19 (2%)
A level/State enrolled/registered Nurse/C&G Final or Full Technical	506 (22%)	834 (37%)	830 (37%)	84 (4%)
Degree level	213 (16%)	492 (37%)	557 (42%)	72 (5%)
**Weekly family income***(n = 8,491)*				
Less than £100	280 (43%)	177 (27%)	174 (26%)	26 (4%)
Between £100 - £199	558 (42%)	446 (34%)	268 (20%)	58 (4%)
Between £200 - £299	758 (34%)	850 (38%)	571 (26%)	54 (2%)
Between £300 - £399	505 (27%)	774 (41%)	528 (28%)	68 (4%)
Greater than £400	465 (19%)	859 (36%)	954 (40%)	118 (5%)

## 4 Discussion

A set of latent classes described parental substance use in a community sample: very low users, low users, moderate users, and heavy users. We found that mothers and their partners had similar consumption profiles for alcohol, but partners consumed more alcohol than mothers, most likely reflecting widely observed sex differences in alcohol consumption as most partners were male. We did not identify a class in which partners were heavy alcohol users and mothers abstain, or vice-versa, but the very low and low user classes had moderate proportions (13–14%) of partners who consumed heavy amounts of alcohol whilst the mother consumed none, or low amounts of alcohol, aligning with behaviours that are found in some families (Templeton, Zohhadi, & Velleman, 2007). This lack of separation in the classes for the partners’ alcohol use could be due to the analysis using mothers’ alcohol use as the principal variable, as it had the greatest number of observations.

Other findings were that as mothers’ alcohol consumption increased across classes so did the proportion of mothers that had said ‘yes’ to using illicit substances in the past year. The moderate and heavy users classes showed higher proportions of mothers engaged in illicit drug use (8% and 20% respectively) compared to the very low and low user classes (4% and 3% respectively). This suggests that those who use alcohol heavily, or use drugs, are more likely to be poly-drug users. This reinforces other research that also finds a co-occurrence of heavy alcohol use and drug use in general population research (Agrawal et al., 2007, Evans-Polce et al., 2016). Subsequently, parental alcohol and drug use at more harmful levels may be correlated and services should consider supporting poly-drug users in addition to distinct alcohol or illicit drug treatment services.

This research has important implications for understanding how parental substance use impacts on child wellbeing. It provides evidence that parents who use greater amounts of alcohol are likely to mirror each other’s use. This means that children may reside in a household where there is no unaffected adult. The implications of this are significant when research suggests that dual-parental alcohol use poses a greater risk for child wellbeing (Berg et al., 2016, Velleman and Templeton, 2016). In addition, the results suggest that parents who consume greater amounts of alcohol had mothers who were more likely to have used drugs in the past year. This finding is key when research has suggested that poly-drug use by parents poses a greater risk for child wellbeing (Raitasalo, Holmila, Autti-Rämö, Notkola, & Tapanainen, 2015). Further research should pay attention to the childhood effects of dual-parental substance use, including poly-drug use, whilst acknowledging that alcohol and/or drug use can occur in isolation.

### 4.1 Limitations and suggestions for future research

This research has several limitations. First, ALSPAC is an opportunistic community sample, where affluent white groups are over-represented, and it is unlikely to be representative of the UK. The data used originates from 1994 to 1997, and views regarding alcohol and drug use, particularly in a family context, have changed due to greater health education and other factors. In addition, the analysis could have been improved if feasible observations regarding the partners’ drug use were available, as it would have given another dimension to understanding parental substance use. It may have also improved if measures of alcohol use for mothers and partners were more similar, as the use of weekly consumption compared to frequency of > 4 units over the month makes it difficult to be certain whether the partners’ use is higher than mothers’ use. In addition to the difference in measures, mothers’ alcohol use is self-reported and may be liable to under-reporting, a bias that may not be as evident when reporting their partner’s alcohol use. Furthermore, the difficulty in identifying a heavy-partner mother-abstainer subtype is likely to be a limitation of the latent class analysis technique, due to the mothers’ alcohol use being the principal variable. Nevertheless, detailed data on parental substance use in the UK are limited, and ALSPAC provides valuable estimates on this issue. Further research should consider replicating this analysis with more recent, representative data to consider whether the findings are, or are not, robust. Furthermore, it should consider the diversity of ‘parents’ and ‘families’ which exist in society i.e. single parents, or grandparents.

### 4.2 Conclusions

This is the first study that has used latent class analysis to understand parental substance use in the United Kingdom. We identified four distinct parental substance use classes and found that parents consume similarly to one another and that a fifth of those who use alcohol heavily are also likely to consume illicit substances. The findings have important implications for how future research should consider the use of alcohol and drugs by parents in terms of service provision, and the effect it may have on child wellbeing. Further research should consider whether these findings are replicated when using other samples, measures, and estimators.

## Funding

This research was funded by an ESRC Wales Doctoral Training Centre (DTC) PhD Studentship at Cardiff University. The work was also undertaken with the support of The Centre for the Development and Evaluation of Complex Interventions for Public Health Improvement (DECIPHer), a UKCRC Public Health Research Centre of Excellence. Joint funding (MR/KO232331/1) from the British Heart Foundation, Cancer Research UK, Economic and Social Research Council, Medical Research Council, the Welsh Government and the Wellcome Trust, under the auspices of the UK Clinical Research Collaboration, is gratefully acknowledged. SCM acknowledges support from the Economic and Social Research Council, the Medical Research Council and Alcohol Research UK to the ELAStiC project (ES/L015471/1). The UK Medical Research Council and Wellcome (Grant ref: 102215/2/13/2) and the University of Bristol provide core support for ALSPAC. This publication is the work of the authors and Emily Lowthian will serve as a guarantor for the contents of this paper.

**Contributors**

EL wrote the manuscript and conducted the analysis. GM and SCM assisted in conceptualising ideas, manuscript preparation, and interpretation of results. Both GG and SMK assisted in the analysis and manuscript preparation.

**Author contribution statement**

1. Emily Lowthian is a PhD Student at Cardiff University. She cultivated the ideas, alongside Graham Moore and Simon Moore, managed the data, analysed the data and wrote the first draft of the manuscript.
2. Graham Moore is a Reader at Cardiff University. He supervises Emily Lowthian’s thesis and helped cultivate the idea for the analysis, aided the interpretation of the analysis and aided the manuscript writing.
3. Simon Moore is a Professor at Cardiff University. He supervises Emily Lowthian’s thesis and helped cultivate the idea for the analysis, aided the interpretation of the analysis and aided the manuscript writing.
4. Giles Greene is a Lecturer at Cardiff University. He is Emily Lowthian’s progress reviewer for her thesis. He co-developed the analysis with Emily, aided the interpretation and manuscript writing.
5. Sara Madeleine Kristensen is a Doctoral Student at the University of Bergen. She aided the development and interpretation of the analysis, and aided the manuscript writing.

## Declaration of Competing Interest

The authors declare that they have no known competing financial interests or personal relationships that could have appeared to influence the work reported in this paper.
