# Supplementary material for: A Latent Class Analysis of Parental Alcohol and Drug Use: Findings from the Avon Longitudinal Study of Parents and Children
Source: Addict Behav. 2020 May;104:106281. doi: 10.1016/j.addbeh.2019.106281 (PMC7378565; doi:10.1016/j.addbeh.2019.106281)
Supplement: Supplementary data 1 [file mmc1.docx]

|  | **Very low users** | **Low users** | **Moderate users** | **Heavy users** |
| --- | --- | --- | --- | --- |
| **Monday** | 0.00 | 0.11 | 0.82 | 3.16 |
| **Tuesday** | 0.00 | 0.12 | 0.88 | 3.01 |
| **Wednesday** | 0.00 | 0.19 | 0.99 | 2.95 |
| **Thursday** | 0.00 | 0.20 | 1.01 | 2.90 |
| **Friday** | 0.00 | 0.66 | 1.59 | 3.44 |
| **Saturday** | 0.00 | 1.29 | 2.08 | 3.82 |
| **Sunday** | 0.00 | 0.59 | 1.28 | 2.92 |

Table A.1: Average number of glasses mothers’ drink per day for each class

|  |  | **Very low users** | **Low users** | **Moderate users** | **Heavy users** |
| --- | --- | --- | --- | --- | --- |
| **Partners’ alcohol use (>4 units)** | **None** | 31% | 16% | 7% | 6% |
|  | **1 - 2 days** | 23% | 22% | 12% | 2% |
|  | **3 - 4 days** | 17% | 25% | 18% | 10% |
|  | **5 - 10 days** | 15% | 25% | 34% | 12% |
|  | **> 10 days** | 10% | 10% | 24% | 33% |
|  | **Everyday** | 4% | 3% | 6% | 37% |
|  |  |  |  |  |  |
| **Mothers’ drug use** | **Yes** | 4% | 3% | 8% | 20% |
|  | **No** | 96% | 97% | 92% | 80% |

Table A.2: Sample proportions for each class for partners’ alcohol use and mothers’ drug use
